# Parents and teachers of children in special education settings value in-school eyecare and written reports of visual status

**DOI:** 10.1371/journal.pone.0238779

**Published:** 2020-09-11

**Authors:** Emma L. McConnell, Shelley A. Black, Julie F. McClelland, Lynne McKerr, Karola Dillenburger, Pamela Anketell, A. Jonathan Jackson, Julie-Anne Little, Kathryn J. Saunders

**Affiliations:** 1 Centre for Optometry and Vision Science, School of Biomedical Sciences, Faculty of Life and Health Sciences, Ulster University, Coleraine, Northern Ireland; 2 Belfast Health and Social Care Trust, Belfast, Northern Ireland; 3 Northern Ireland Clinical Research Facility, Belfast City Hospital, Belfast, Northern Ireland; 4 School of Social Sciences, Education and Social Work, Queen’s University, Belfast, Northern Ireland; University of Botswana Faculty of Medicine, BOTSWANA

## Abstract

**Objectives:**

To evaluate parent and teacher opinion of the provision of in-school eyecare and jargon-free written reporting of visual status for children in special educational settings.

**Participants and methods:**

A nationally-agreed, in-school eyecare framework for children attending special schools which recommends a full eye examination, dispensing of spectacles and provision of a jargon-free written report of visual outcomes to parents and teachers, was provided to 200 children (mean age 10 years, 9 months; 70% male) attending a special school in the UK. The written ‘Vision Report’ detailed, in lay-language, results from the eye examination and provided practical advice to alleviate the impact of vision difficulties both at home and in the classroom. Following implementation of the framework, parents and teachers completed a feedback questionnaire to determine their opinion of the in-school eye examination and utility of the Vision Report.

**Results:**

Parents of 123 participants returned a feedback questionnaire. Eighty-eight participants were represented by the 23 teachers who returned a questionnaire. The in-school eyecare was rated positively for children in special education by 82.4% of parents and 80.9% of teachers. Key benefits included the familiarity of the in-school setting (81.3% of parents and 100% of teachers agree), the convenience of the setting for parents (74.0% of parents and 100% of teachers agree), and the opportunity for teachers to speak directly to eyecare providers regarding a child’s visual needs (82.6% of teachers agree). The information provided by the Vision Report was deemed useful day-to-day by 78.3% of parents and 100% of teachers. The majority (80%) of teachers implemented classroom modifications suggested in the report, whereas only 47.9% of parents reported implementation of modifications at home.

**Conclusions:**

Provision of in-school eyecare is valued by parents and teachers of children in special education settings. Jargon-free, written reports of visual status are valued and utilised by parents and teachers. Further support is required to aid parents in implementing vision modifications at home.

## Introduction

It is well established that children with developmental disability are at an increased risk of visual problems compared to their typically developing peers [1–4]. Despite the increased risk of visual deficits, this vulnerable group of children are often reported as having poor history of eyecare [5]. This finding indicates that barriers exist for children accessing eyecare services. Barriers, including parental concern regarding the child’s ability to cooperate with testing procedures, fear of behavioural difficulties as a consequence of waiting times at appointments and difficulty accessing clinic appointments if the child has a physical disability, have previously been reported by parents of children with developmental disability in relation to other aspects of healthcare [6–9].

Even when barriers to accessing eyecare are overcome, outcomes from a visual assessment are often verbally communicated to parents, meaning that key information may be forgotten or misunderstood [10, 11]. This reduces the likelihood of appropriate management strategies being implemented in the child’s home or school environment, particularly where information is not shared with teachers or educators. Impaired academic performance has been associated with visual deficits, in both mainstream and special education settings [12] and correction of visual deficits has a positive impact on classroom behaviour [13]. It is therefore important that meaningful information on visual status and visual need is effectively translated and delivered to all stakeholders involved in a child’s care, including educators [14].

In mainstream education settings, orthoptic-led vision screening is recommended at school entry level by the United Kingdom National Screening Committee [15] to identify reduced vision and initiate prompt treatment, thus minimising the risk of permanent vision loss from conditions such as amblyopia. Public Health England’s guidance is that while school entry vision screening is recommended for children in mainstream education, it is not appropriate for children attending special schools who should have access to comprehensive in-school eyecare, including full eye examinations and on-site dispensing of spectacles where required from a special school ophthalmic team [16]. A key component of this guidance is that outcomes from the eye examination are communicated to parents, teachers and other stakeholders involved in the child’s care in the form of a report written in lay-language.

Previous work from our group evaluated whether the implementation of comprehensive eyecare in special educational settings, as described in the sector-agreed framework published by the Royal College of Ophthalmologists [16], had measurable benefits for children in terms of their visual status, behaviour and engagement in the classroom, and how well significant visual deficits were addressed or compensated for in the home and school environment [13]. The present study aimed to evaluate the benefit to parents and teachers, of (i) providing children in a special education setting with a comprehensive in-school eye examination and (ii) providing a lay-language ‘Vision Report’ to stakeholders involved in the child’s care.

## Methods

**Fig 1:**
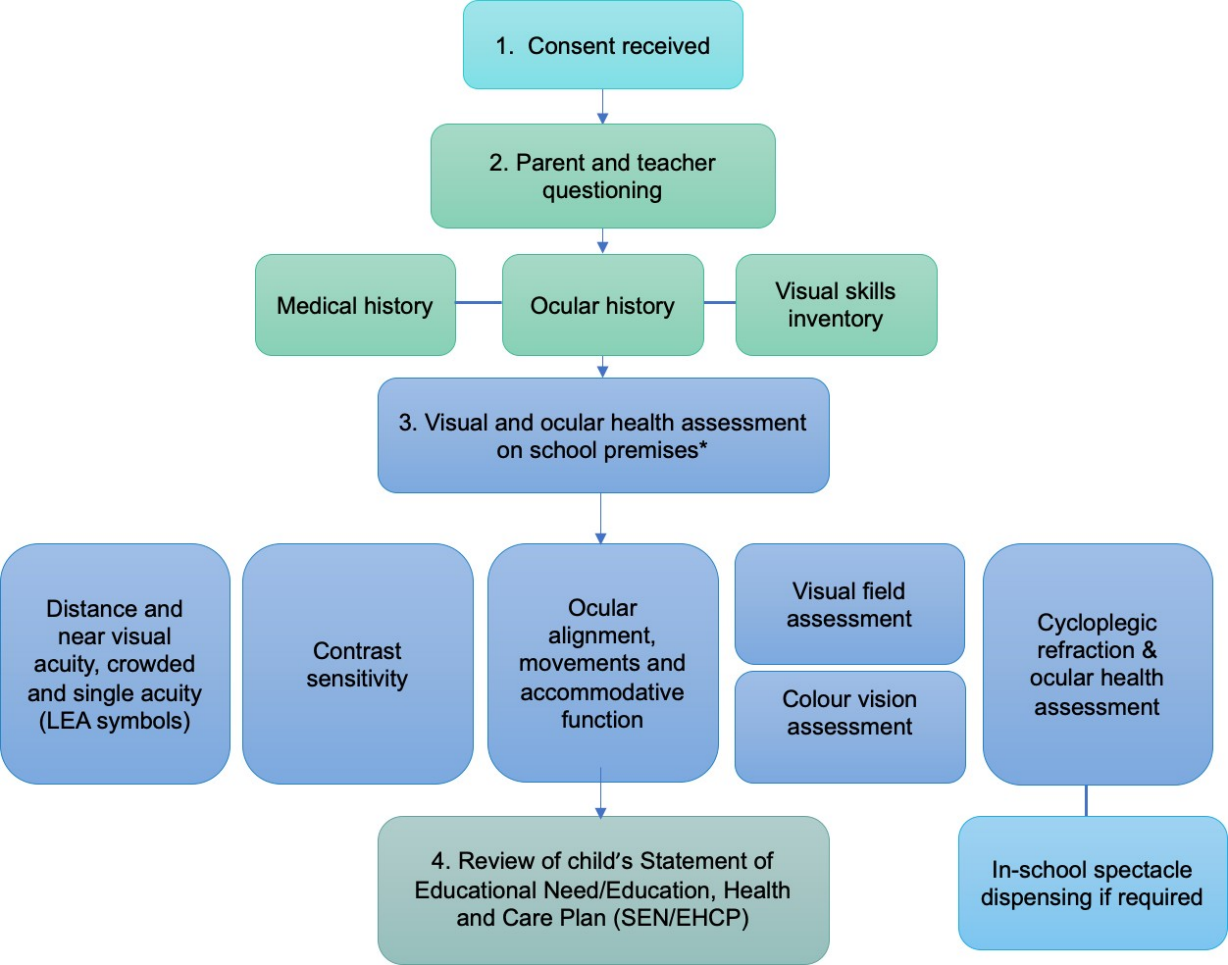
Summary of in-school assessment process and procedures. *Tests suitable for the child’s age and ability were employed. The vision assessment was carried out over several visits to aid compliance if required.

Ethical approval for this study was gained through Ulster University Research Ethics Committee (REC/15/0125) and the study adhered to the Tenets of the Declaration of Helsinki. Detailed methods for this school-based observational study have been described previously (see Black et al. 2019 [13]) and are summarised in Fig 1. In brief, parents of all pupils attending the largest special school in Northern Ireland, which reported receiving no previous in-school vision assessment or screening, were invited to enrol their child or young adult in the study for a comprehensive vision assessment (see Fig 1), in-school spectacle dispensing (where necessary), and provision of a Vision Report. Vision assessment procedures and tests employed to ascertain visual status are detailed in Table 1. A range of tests were selected to suit the individual needs of each child. Parents and teachers completed questionnaires providing information about each child/young adult. The profile of the school’s pupils’ learning difficulties was primarily moderate (n = 141) or severe (n = 104).

**Table 1:** Vision assessment procedures and methods employed to ascertain visual status. A test suitable for the child’s ability was selected.

Vision assessment		Method
Vision	Distance	Sonksen crowded LogMAR letters
		LEA crowded LogMAR symbols
		LEA single LogMAR smbols
		Cardiff Acuity Test
		Bradford Visual Function Box
	Near	Sonksen crowded LogMAR letters
		LEA crowded LogMAR symbols
Ocular alignment		Prism cover test at distance and near
Ocular movements		Ocular movements in eight directions of gaze
		Near point of convergence
		Quality of pursuit and saccadic eye movements to penlight at 40cm
Accommodative function		Dynamic retinoscopy with Ulster-Cardiff accommodation cube with target at 25cm/4D
Contrast sensitivity		Cardiff Contrast Test
Visual field		Binocular gross confrontation to 5cm white ball
Refractive error		Cycloplegic retinoscopy (1% cyclopentolate HCl)
Ocular health		Direct/indirect ophthalmoscopy
Visual processing		Crowded acuity ratios using LEA crowed and single logMAR symbols
		Visual Skills Inventory completed by parents

### Vision report

**Fig 2:**
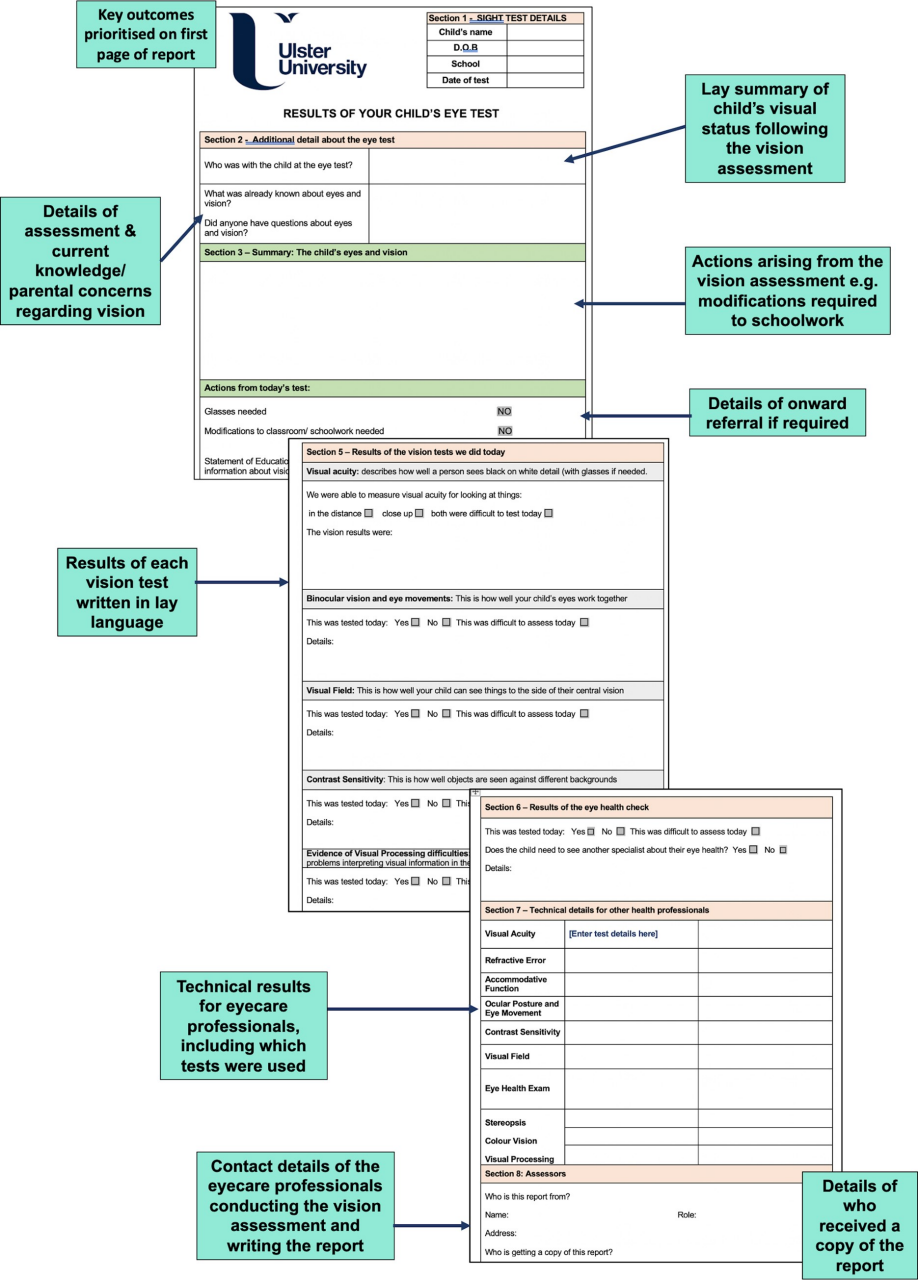
Information detailed in the Vision Report [17] following in-school vision assessment.

Following the comprehensive vision assessment, parents and teachers were provided with a structured Vision Report. This report was written by the clinicians undertaking the visual assessment. In addition to reporting technical information about visual status suitable for sharing with other eye/medical professionals, findings were presented in lay-language. Reports offered practical advice on how to account for any vision difficulties the child/young adult presented with. The Vision Report proforma used in the present study was developed by Ulster University and SeeAbility [17] and modified following feedback from a previous in-school pilot study conducted during March-June 2016. Fig 2 details the information included in the Vision Report. An example report is available in online supporting information (S1 Fig).

Within each section, if a child/young adult had a ‘normal for age’ result, parents were informed of this positive finding. Normal results were defined using previously published normative data for each test used [13]. Where reduced function was identified, this was communicated and practical advice provided to mediate the impact of this deficit, where appropriate. For example, if the child had a restriction of their visual field on their right-hand side, advice regarding seating position, placement of school work, food etc. was provided to maximise use of the child’s available vision. If a child demonstrated an accommodative deficit, provision of bifocal or near work spectacles was implemented along with detailed advice on when these spectacles should be worn. Where a child was under the care of another eyecare provider, written information was provided to this individual regarding the findings and outcomes of the in-school eye examination.

The Vision Report was complemented with additional information from the Ulster Vision Resources (UVR) where required [18]. The UVR is an online tool previously developed by eyecare clinicians and researchers at Ulster University to aid parent and professional understanding of a child’s vision difficulties. Amongst other resources, the UVR provides downloadable examples of suitable images and font sizes for children with reduced visual acuity at distance or near. The UVR also contains advice for parents on how to encourage children to wear their spectacles if compliance is an issue. Printed resources were provided to parents and teachers as required.

### Parent and teacher feedback

A feedback questionnaire was developed to determine the value to parents and teachers of the in-school vision assessment and Vision Report. Two versions of the questionnaire were developed after piloting; one for parents and one for teachers (S2 and S3 Figs). Both questionnaires included a variety of 5-point Likert scale questions, yes/no and free text responses. The project was completed across two academic years (2016/7 and 2017/8) and, as such, feedback questionnaires were distributed to all teachers upon completion of the project at the end of the second year. Some teachers had more than one pupil participating in the project, however they completed only one questionnaire to cover their experience of the in-school eyecare. Parents were provided with a single questionnaire at the end of the academic year during which their child received an in-school eye examination. Parent and teacher questionnaires contained 16 and 13 items respectively to identify benefits and limitations of the in-school eye examination and their perception of the utility of the eye examination and Vision Report for parents, school staff and pupils.

### Statistical analysis

All statistical analyses were performed using IBM SPSS Statistics for Macintosh (Version 25.0, Armonk, NY:IBM Corp 2017). Descriptive statistics were used to summarise parent and teacher responses from the questionnaire items. The Shapiro-Wilk test was applied to determine normality of the test data and as such non-parametric and parametric tests were applied where appropriate. Mann-Whitney U tests were applied to compare differences in parental responses between different eyecare services. A p value of <0.05 was considered statistically significant.

## Results

### Participant profile

Parental consent to receive the in-school eyecare was obtained for 200/335 (59.7% response) pupils attending the school across two academic years from September 2016 to June 2018. Participants ranged in age from 3 years 7 months to 19 years 9 months (median 10 years 10 months; mean 10 years 9 months). Seventy percent were male (n = 140); representative of the special educational needs population in Northern Ireland [19].

Participants presented with a range of medical conditions and syndromes including Autism spectrum disorders (ASD;n = 67, 33%), Down syndrome (n = 18, 9.7%) and cerebral palsy (n = 5, 2.7%). Review of the child’s Statement of Educational Need/Education, Health and Care Plan (SEN/EHCP) revealed 70 (35%) pupils were reported as having Severe Learning Difficulties (SLD), 80 (40%) Moderate Learning Difficulties (MLD), one (0.5%) Mild/Moderate and two (1%) Profound Learning Difficulties. Children with Down syndrome were significantly more likely to have SLD (Chi-square p = 0.002). No such associations were evident for children with ASD or cerebral palsy (p = 1.000 and p = 0.187 respectively). Participants represented the profile of the school in terms of level of learning difficulty, gender and age (Chi-square p = 0.722 for learning difficulty; p = 0.446 for gender; Mann-Whitney U p = 0.053 for age). Testing procedures were adapted to suit the needs of each individual child, for example, assessments were conducted in the child’s classroom if they felt most comfortable there; a calm, controlled approach was undertaken for children with ASD if preferred; postural support was utilised for children with cerebral palsy where required. Binocular visual acuity ranged from -0.30 to 1.30 logMAR (median 0.00logMAR) and spherical equivalent refractive error ranged from -14.00 to +9.25 dioptres (median +0.75 dioptres). Three (1.5%) children had a certification as Severely Sight Impaired or Sight Impaired; one child had congenital cataracts, one traumatic brain injury and for one the cause was unknown. Parents of these children reported receiving additional help from vision support services (Qualified Teacher of the Visually Impaired (n = 1; 33%) or vision support charities (n = 2; 67%)). The majority had a visual profile which did not qualify for certification or additional support, although 8.6% (n = 17) were identified as having reduced vision at distance and/or near compared with age normative data. Sixty-three (32%) children had significant refractive errors and 33 (17%) exhibited hypo-accommodation. Twenty-four (13.1%) children presented with reduced contrast sensitivity, four (2%) had gross visual field deficits, 39 (19.5%) had a manifest strabismus at distance, near or both, and nine (4.5%) had nystagmus. Forty-three participants exhibited evidence of visual processing difficulties and five had a colour vision deficit. Eighteen (9%) children had one or more ocular health anomalies. More detailed visual profile of these participants has been published previously [13].

### Feedback questionnaire return rates

#### Parent

Parent/guardians of 196 children consented to receive a feedback questionnaire; 123 (62.8%) were returned to the research team. The profile of participants for whom a questionnaire was returned was comparable with those for whom a questionnaire was not returned in terms of level of learning difficulty (Fisher’s exact test p = 0.110) and age (Mann Whitney U = 4040.50, p = 0.242). Results presented below are representative of the 123 children or young adults for whom a questionnaire was returned, rather than the entire study population.

#### Teacher

Forty teachers were invited to complete a feedback questionnaire; 23 were returned (57.5%). Some teachers were responsible for multiple pupils who were participants in the study, while others taught only one pupil involved in the study. Teachers were asked to complete one questionnaire only, representing all pupils in their class who participated rather than one questionnaire per pupil. A total of 88 pupils were represented by the teacher’s responses (44.0%).

### In-school eyecare service feedback

#### Usefulness of the in-school service

**Fig 3:**
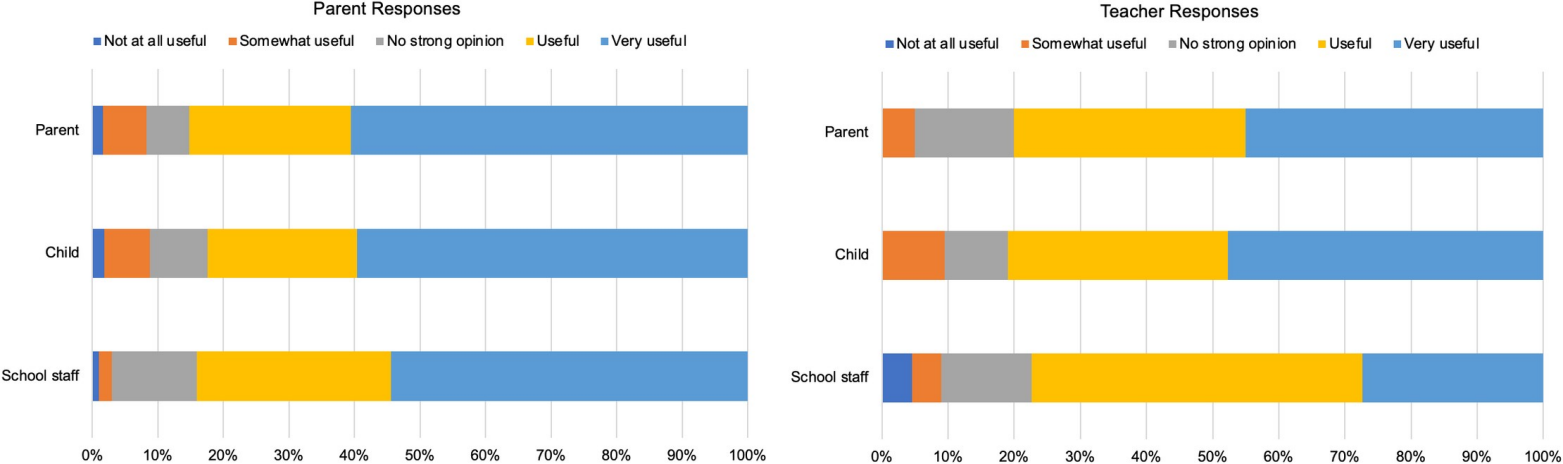
Parent and teacher opinion on the usefulness of the in-school vision assessment for themselves and for the child.

Both parents and teachers were questioned on the utility of the in-school vision assessment for themselves and for the children (Fig 3). The majority of parents and teachers reported positively; 84.6% of parents (n = 104) rated the in-school vision assessment as ‘very useful’ or ‘useful’ for themselves, and 84.2% reported the same for their child’s teacher. Two parents (1.6%) reported the vision assessment was ‘not at all useful’ for them. From the teacher perspective, 77.3% of teachers reported the vision assessment as useful/very useful for them, and 80% felt it was useful/very useful for parents. One teacher and one parent rated the eye exam as ‘not at all useful’ for school staff. With regard to the utility of the in-school vision assessment for the children, 82.4% of parents and 80.9% of teachers reported ‘useful’ or ‘very useful’. All staff reported positively (i.e. responses of ‘very useful’, ‘useful’ or ‘somewhat useful’) on the usefulness of the in-school eye examination for the children, whereas two parents rated it as ‘not at all useful’ for their child.

#### Benefits and limitations of the in-school eyecare service

**Fig 4:**
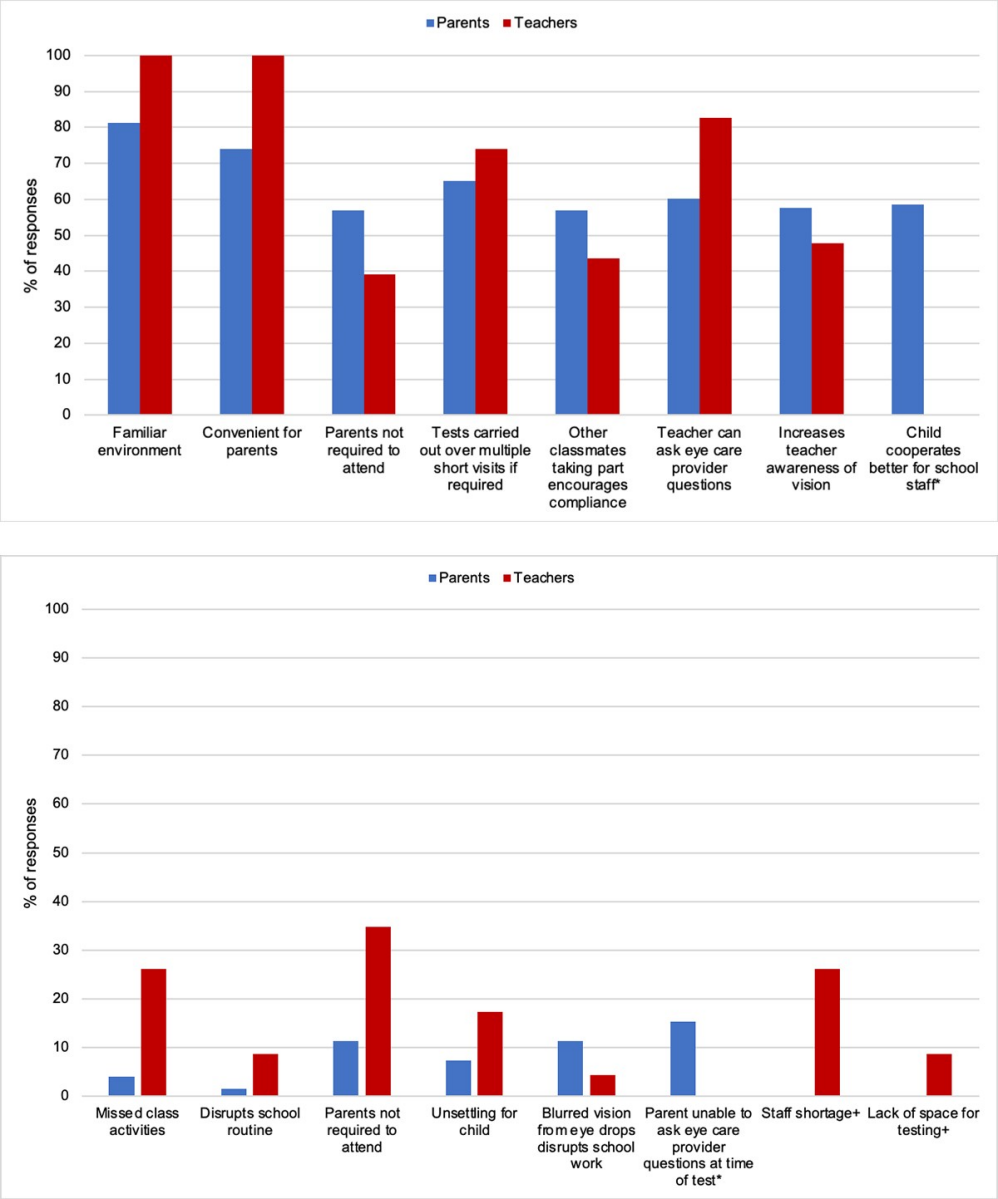
In-school eye examination a) benefits and b) limitations reported by parents and teachers. *Question asked to parents only, +question asked to teachers only.

Parents and teachers were asked to identify, from a selection of offered statements, the benefits and limitations they perceived in the in-school vision assessment service. Several statements could be selected and there was opportunity for additional free text responses. Results are shown in Fig 4A and 4B. For parents, the most commonly reported benefits were the familiarity of the school setting (81.3%), the convenience of having the vision assessment completed in school (74.0%) and that the assessment could be carried out over multiple short visits if the child required, for example due to challenges in compliance or attention (65.0%). All teachers responded that the familiarity and convenience for parents were of benefit, while 82.6% reported that being able to speak directly to the eyecare providers regarding a child’s vision and visual needs was beneficial. Free text responses from parents centred around the benefit of the in-school setting in minimising time away from school to attend external appointments, and the added convenience this has for working parents. Seventeen teachers (73.9%) reported the vision assessment did not disrupt the pupil’s other school activities.

#### Limitations were reported less frequently

For parents, the most commonly identified limitation (reported by 15.4%) was the inability to speak to the eyecare provider directly at the time of testing. Similarly, the most common limitation reported by teachers (34.8%) was that the parents may not be present at the eye examination, however, nine teachers (39.1%) considered this beneficial. All parents were offered the option to attend the vision assessment, however few availed of this. Other limitations reported by teachers related to the child missing class activities to attend the eye examination and staff shortages to accompany the child to their examination (both 26.1%).

#### Provision of spectacles

In line with the sector-agreed framework under test [16], where new or updated spectacles were required, in-school dispensing of spectacles was offered. We asked parents whether, if their child required spectacles, they had a preference on how these were dispensed. A total of 116 parents responded to this question, with some parents choosing more than one option. The majority of parents (56.9%, n = 66) reported they would be happy for their child to have spectacles chosen, dispensed and fitted at school. Of these, 52 (78.8%) reported they would like to be involved in choosing the frames, while the remaining 14 (21.2%) reported they were comfortable not being involved in frame choice. Thirty-three parents (28.4%) reported they would prefer to get their child’s spectacle prescription dispensed in their local opticians/optometrists, and 22 parents (19%) reported they had no preference for where spectacles were dispensed.

#### Parent opinion regarding in-school eyecare compared with previous eyecare services

**Fig 5:**
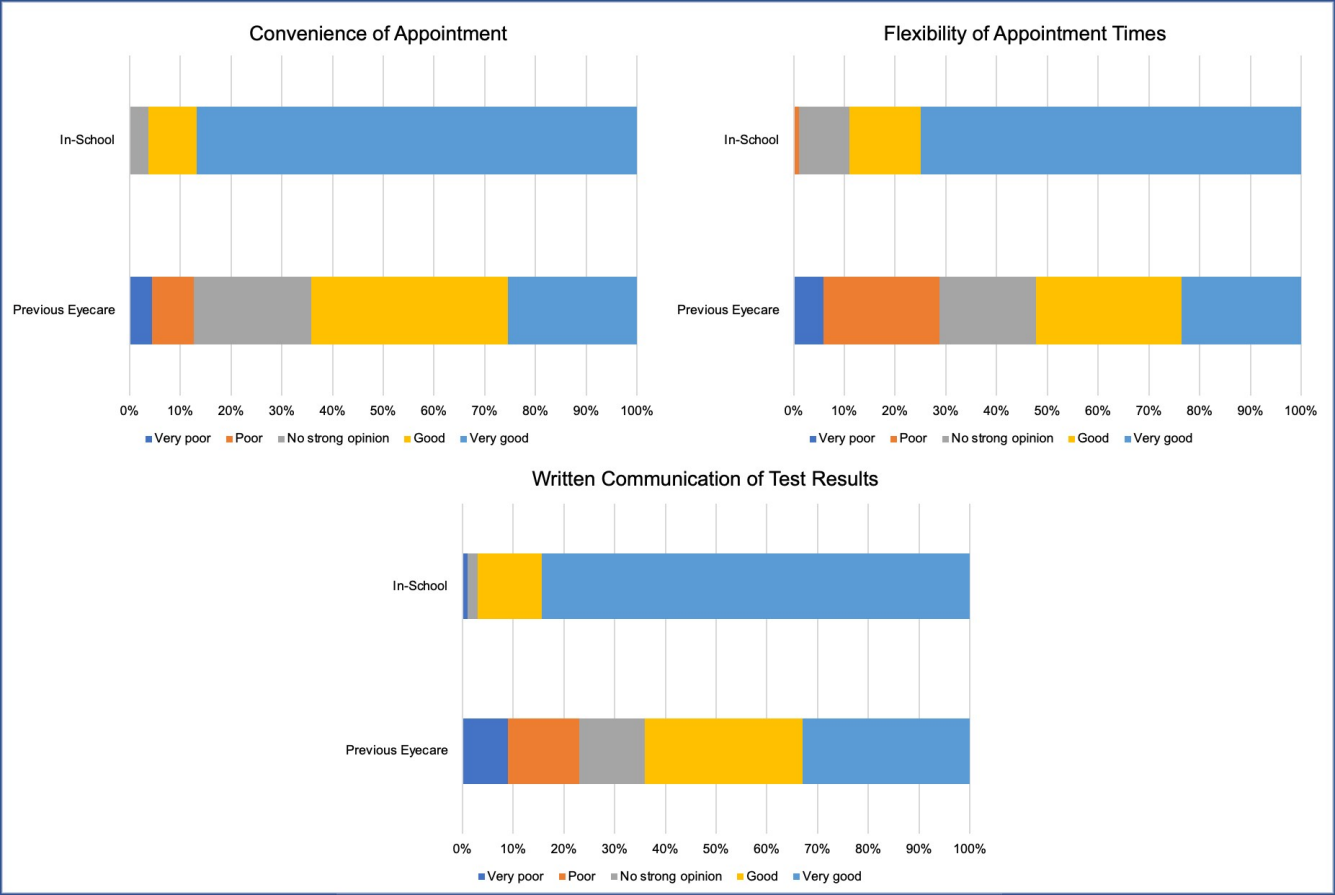
Responses from parents regarding their experience of various aspects of in-school eyecare and previously accessed eyecare services (hospital or community based).

Parents were asked to rate their experience (see Fig 5) of the in-school eyecare and eyecare services their children had previously accessed i.e. hospital eye service and/or community eyecare at optometrists/opticians. Three items were explored using a five-point Likert scale with options ranging from ‘very poor’ to ‘very good’. Not all children had previous history of eyecare, therefore results are representative of parents who answered each question (n = 118 for in-school eyecare, n = 61 for previous eyecare services). To determine whether responses differed significantly, Mann-Whitney U tests were carried out. There was a statistically significant difference between responses for all items, with in-school eyecare ranking more positively for experience across all aspects (p<0.001 for all).

### Vision Report feedback

#### a) Parent feedback on Vision Report

*Actions provided requiring parental input*. Of the 123 parents who returned a questionnaire, an action which required parental input was recommended for 71 participants (57.7%). The number and type of these actions are shown in Table 2. Some children required more than one action. The most common reason for advice was to highlight issues relating to visual processing difficulties (n = 41). Such difficulties were identified either by comparing crowded vs uncrowded acuity scores or from examination of parental responses to the Visual Skills Inventory questionnaire. Provision of new/updated spectacles was the second most common action (n = 20).

**Table 2:** Number and type of actions provided to parents (n = 71) and teachers (n = 70) in Vision Report (only includes the 123 children represented by parents who returned a feedback questionnaire).

Interventions/actions	Requiring parent action, n (% of those provided with actions in report)	Requiring teacher action, n (% of those provided with actions in report)
**Advice to account for crowding or visual processing deficits**	41 (57.7)	40 (57.1)
**Implementing new/updated spectacle wear**	20 (28.2)	20 (28.6)
**Enlarge print size of written material**	13 (18.3)	13 (18.6)
**Increase contrast of written/play material**	12 (16.9)	12 (17.1)
**Provided strategies to encourage spectacle wear**	12 (16.9)	12 (17.1)
**Advice to account for visual field deficit**	2 (2.8)	2 (2.9)
**Additional assistance required to maintain place when reading due to eye tracking problems**	1 (1.4)	1 (1.4)
**Referral to GP (n = 2) or hospital eye service (n = 4)**	6 (8.5)	Teacher input not required
**Convergence exercises prescribed**	3 (4.2)	Teacher input not required
**Blepharitis management**	1 (1.4)	Teacher input not required
**Account for colour vision deficit in class**	Parent input not required	5 (7.1)

*Implementation of suggested actions at home*. Where an action had been suggested in the Vision Report, parents were asked to feedback whether they had implemented, or planned to implement, these modifications in the child’s home environment. Of the 71 parents who were provided with an action requiring parental input, 34 (47.9%) reported they had implemented the necessary modifications at home, 11 (15.5%) reported they had not, three (4.2%) answered that they did not know and 15 (21.1%) reported that this question was not applicable to them despite having been provided with actions in the report. Eight parents (11.3%) did not provide a response for this question. The number and type of actions suggested to parents along with their response to whether this had been implemented or not are shown in Table 3. Aside from management of blepharitis (recommended for one child), the most commonly implemented action (70%, n = 14) related to new or updated spectacle prescription. Strategies to encourage spectacle compliance were implemented by 58.3% of parents (n = 7) for whom this was suggested. Actions which required environmental modifications to be made to the child’s home environment, work or play material were less frequently implemented, for example increasing print size (46.2%) or contrast (50.0%) of written material, or practical strategies to account for visual processing difficulties (48.8%).

**Table 3:** Responses to parent implementation of suggested actions in Vision Report.

Interventions/Actions	Action implemented at home						
	Yes	No	Don’t know	Not applicable	Total not implemented	Not answered	Total
**Advice to account for crowding or visual processing deficits**	20 (48.8%)	7	0	10	17 (41.5%)	4	41
**Implementing new/updated spectacle wear**	14 (70%)	3	1	1	5 (25%)	1	20
**Enlarge print size of written material**	6 (46.2%)	2	1	1	4 (30.8%)	3	13
**Increase contrast of written/play material**	6 (50%)	0	2	4	6 (50%)	0	12
**Provided strategies to encourage spectacle wear**	7 (58.3%)	1	1	1	3 (25%)	2	12
**Referral to GP (n = 2) or hospital eye service (n = 4)**	0 (0%)	1	0	2	3 (50%)	3	6
**Convergence exercises prescribed**	1 (33.3%)	0	0	1	1 (33.3%)	1	3
**Advice to account for visual field deficit**	0 (0%)	1	0	0	1 (50%)	1	2
**Blepharitis management**	1 (100%)	0	0	0	0	0	1
**Additional assistance required to maintain place when reading due to eye tracking problems**	0 (0%)	0	1	0	1 (100%)	0	1

Of the 34 parents who reported implementation of suggested actions, 23 (67.6%) specified what modifications they had made. Nine parents reduced clutter in the child’s home, nine reported their child had increased compliance with spectacles, two children were provided with enlarged print material and one parent reported changing the home environment to reduce the impact of visual processing difficulties. One parent reported that her child already makes the suggested modifications herself by moving closer to objects to account for reduced vision. Another stated that, while they haven’t currently acted to modify their child’s environment, the Vision Report has made them aware of the child’s vision needs and they plan to make adaptations as the need arises. Of the parents who had not implemented suggested actions, two provided additional comment and both related to spectacle compliance; *“my daughter is non-compliant with wearing glasses*” and *“my son refuses to wear his glasses at school and home*.*”*

*Actions provided requiring teacher input*. Of the 123 parents who returned a feedback questionnaire, 70 were provided with advice in the report which required action at school. The type and number of these actions are provided in Table 2. As with the actions requiring parental input, the most common reason for advice requiring teacher input was to highlight issues relating to visual processing difficulties (n = 40) and implementation of new/updated spectacles (n = 20).

*Parental awareness of implementation of suggested actions requiring teacher input at school (n = 70)*. Parents were asked whether suggested actions had been implemented at school. Fifteen parents (21.4%) reported these had been implemented at school. The majority of parents (n = 34, 48.6%) reported that suggested actions had not been made, or they were unaware whether actions had been implemented. The remaining parents felt this question wasn’t applicable to them (n = 12, 17.1%) or did not respond (n = 9, 12.9%) to this question.

*New information provided to parents*. Parents were asked whether the Vision Report contained any information regarding their child’s eyes and vision which was previously unknown to them. Thirty-six (31.6%) reported they received new information, 64 (56.1%) reported no new information was received and 14 (12.3%) were unsure whether the report provided them with new information. Of the 36 parents who received new information regarding their child’s vision, 31 (86.1%) had a previous history of eyecare. Thirty-three parents provided written comment regarding what information was previously unknown to them. Information regarding refractive error status was the most commonly reported ‘new information’ (n = 8; spectacles provided for the first time in four of these cases), closely followed by knowledge of what level of vision their child had (n = 6). The latter was not always in relation to a reduced visual acuity as some parents commented that it was reassuring to know their child had a good level of vision. Five children were identified as having a colour vision defect in the study population. Parents of four of these children reported that they were previously unaware of their child’s colour vision problem; all had a previous history of eyecare. Three parents provided more general comments on the information contained in the report: one commented that their child had never had a conclusive eye test before indicating that all of the information contained in the report was new to them; one remarked that they appreciated that the report *“gave specific findings*”; and one parent was aware of new information which resulted in changes to her classroom environment.

*Parent-reported usefulness of written reports*. Parents were asked whether they found the information contained in the Vision Report useful on a day-to-day basis using a Likert scale of responses ranging from ‘not at all useful’ to ‘very useful’. Of the 123 parents who returned the questionnaire, 115 responded to this question. The majority of these parents (78.3%) found the information in the Vision Report either ‘very useful’ or ‘quite useful’ on a day-to-day basis. To determine if parents who found the information contained in the report useful on a day-to-day basis were more likely to have implemented actions suggested in the Vision Report, responses were grouped into positive and negative response categories. Responses of ‘very useful’, ‘quite useful’ or ‘parts are useful’ were ranked as positive while responses of ‘no strong opinion’ or ‘not at all useful’ were ranked as negative. Parent report of actions implemented at home were also assigned into two groups; ‘yes’ or ‘no’. The latter included responses of ‘no’, ‘don’t know’ or ‘not applicable’. Of the 71 parents who received an action for their child, 62 answered both these questions and were included in this analysis. Fifty-five parents had a positive response to the utility of the report; thirty-three (53.2%) had implemented the suggested actions at home, compared with 22 parents who had not yet implemented these. Six parents (9.7%) who had a negative response to the usefulness Vision Report had not implemented suggested actions, while one other parent in this category (1.6%), despite reporting that the Vision Report was not useful had implemented the actions the Report had recommended. Fisher’s exact test showed that parents who found the report useful were significantly more likely to have implemented the suggested actions (p = 0.039).

#### b) Teacher feedback on Vision Report

Of the 23 teachers who returned a feedback questionnaire, 15 (65.2%) reported they received a copy of the Vision Reports for a child/children in their class; thirteen of whom read the reports immediately upon receipt, and two reported reading the report several weeks later.

*Actions requiring teacher input*. Of the 88 pupils represented by the teachers who returned a questionnaire and had participating pupils in their form class, 46 (52.3%) children’s Vision Reports recommended a vision-related action from their teacher. The number and type of actions provided for the entire pupil population represented by returned teacher questionnaires are shown in Table 4. In addition, this table shows the number and type of actions recommended to the 15 teachers who reported receiving the Vision Report, and thus had a chance of being implemented. Of the 77 pupils represented by the teachers who received the reports, 41 (53.2%) required a teacher action.

**Table 4:** Number and type of interventions represented by teacher feedback questionnaires grouped by total received and teachers who reported receipt of Vision Report.

Interventions/actions	Number of actions recommended to teachers with participating children in class (n = 18)	Number of actions recommended to teachers with children in class who received report (n = 15)	Actions reported as implemented by teachers
**Advice to account for crowding or visual processing deficits**	27	25	22
**Implementing new/updated spectacle wear**	10	9	7
**Enlarge print of written material**	8	7	7
**Increase contrast of written/play material**	10	8	7
**Provided strategies to encourage spectacle wear**	7	5	4
**Account for colour vision deficit in class**	2	2	2
**Advice to account for visual field deficit**	3	2	2
**Additional assistance required to maintain place when reading due to eye tracking problems**	2	2	2

*Implementation of suggested actions at school*. Of the 15 teachers who reported receiving and reading the Vision Report, 12 (80%) had implemented the suggested actions in the report to account for a child’s visual deficit in the classroom. Two teachers reported that they were unsure if modifications had been made: one of these teachers no longer taught the pupils for whom they had received the Vision Reports and the other had received Reports that did not recommend any actions.

Three teachers provided written comments on the modifications they had instigated for pupils in their class. One reported that the Vision Report drew attention to a pupil’s need for high contrast print/written material, and their difficulty discriminating pictures and words if they were a similar colour to the background. In response, the teacher had made adjustments for this; ensuring the child uses a thick, dark pen when writing, and that reading material is presented in a clear, uncluttered manner. Another teacher commented that the child’s *“desk is less cluttered and larger font is used*.*”* One teacher commented that they had introduced a modified *“seating position for the child”* and altered *“font size and type for worksheets”* following receipt of the Vision Report.

*Teacher-reported usefulness of written reports*. Teachers were asked whether they considered the information contained in the Vision Reports useful and relevant to their work with the pupils; 100% responded positively to this question. Four (26.7%) rated the information as ‘very useful’, six (40.0%) ‘quite useful’ and five (33.3%) thought ‘parts were useful’. While most teachers (80.0%) reported they felt confident implementing actions suggested in the report, 60% reported they were interested in further training on how to adapt a child’s environment if they presented with a vision deficit.

*Was the information in the report truly ‘jargon-free’*?. A key aim of the Vision Reports was to ensure they were written in layman’s terms, avoiding technical jargon and providing meaningful, actionable information to non-professional readers. In order to ascertain whether this aim was successfully achieved, we asked parents and teachers to rate whether the reports were written in a way they could understand using a five-point Likert scale ranging from ‘difficult to understand’ to ‘easy to understand’. The majority of parents (80.4%) found the information in the report either ‘easy’ or ‘fairly easy’ to understand (n = 60 and 39 respectively). Ten parents had no strong opinion and four reported the language used was ‘somewhat difficult’ to understand. Likewise, the majority of teachers (93.3% of those who received and read the reports) found the information contained in the report ‘easy’ or ‘fairly easy’ to understand (n = 10 and 4 respectively). One teacher had no strong opinion. Neither parents nor teachers reported that the information in the report was ‘difficult’ to understand.

### Vision information on statutory educational documents

Review of each child’s SEN/EHCP revealed that information relating to vision was included for only 26 (26.5%) of the 98 children for whom the in-school eye examination identified that classroom modifications were required to mitigate against visual deficits. Where information relating to vision was included in the SEN/EHCP, it often stated the child had a squint or they were required to wear spectacles. In many instances, information provided was littered with technical jargon and offered minimal specific advice to aid teachers and educators in successfully accounting for a vision difficulty in the classroom, for example *“Pupil has a moderate alternating convergent squint*, *visual acuity 6/48 with both eyes and rotatory nystagmus*. *She did wear glasses well but is now quite aware of sensations around her face and pulls them off rapidly*.*”* The objective listed in the SEN/EHCP to account for this child’s difficulties was to *“take account of her visual difficulties by using an appropriate structured and learning environment*.*”* No further information was provided to elucidate what constituted “an appropriate structured and learning environment” for this child. Despite the low representation of vision information in children’s statutory documents, 94.1% of teachers reported that, where a visual problem exists, this should be reflected in a child’s SEN/EHCP. Likewise, the majority of parents (n = 83, 73.5%) considered it important to include vision information within this document.

## Discussion

Public Health England recommend that children attending special schools should receive comprehensive in-school eyecare from a special school ophthalmic team. This follows guidance published by the Royal College of Ophthalmologists and other professional eyecare bodies in the UK [16]. Application of the sector-agreed model has been shown to have measurable benefits in terms of reducing a child’s unmet visual needs and increasing classroom engagement [13]. The present study reports that parents and teachers are strongly in favour of comprehensive in-school eyecare provision, further supporting the value of such services for this vulnerable group.

### In-school service

Parents reported preference for in-school eyecare compared with previously accessed eyecare services. A key benefit of in-school eyecare which was repeatedly reported by parents and teachers was the familiarity of this setting for the children. It is well recognised that children with developmental disability, particularly those with autism, prefer routine and familiarity [20]. Attending clinical appointments unfamiliar environments can cause increased anxiety and behavioural difficulties in those with developmental disability [7, 9]. In the present study, with regard to previously accessed eyecare services, one parent commented that the *“very clinical and new surroundings instantly puts my son on edge*,*”* while another stated *“the clinic environment was daunting for my son*.” Providing eyecare in a familiar environment, is likely to reduce stress and anxiety, therefore increasing the likelihood of compliance with testing procedures, ultimately providing more meaningful results. This benefit was frequently voiced by parents, e.g.; *“It is very useful when all happens in a friendly environment like school”*, *“my son was settled in a familiar place and cooperated well*.*”* One parent commented that they were “*always reluctant to take my son to the opticians*,*”* indicating perceived barriers to accessing traditional eyecare services. When a child’s compliance at an examination is poor, more frequent review appointments are required to allow completion of a conclusive eye examination. This creates a compounding burden on already strained NHS services by increasing clinic waiting times and reducing the number of appointments available for new patients. Furthermore, it has been shown that children have a high ‘did not attend (DNA)’ rate at hospital clinics [21, 22]. Provision of eyecare in-school allows for another child to be examined in lieu of any absentees, reducing the financial impact of missed appointments [23].

Two parents reported that the in-school service was ‘not at all useful’ for them or their child. Of these, one parent provided written comment which stated that having an eye examination conducted in school was more convenient and reported that their child behaved better at school, indicating that they did perceive the service as beneficial despite providing a negative response to this question. The child of the other parent who reported the in-school service was not useful did not have significant vision problems requiring advice or intervention which may account for this parent’s responses.

Views on the value of the in-school eyecare service were not available for all parents and teachers as some failed to return a feedback questionnaire (n = 73 parents and n = 17 teachers); therefore, opinion of these individual has not been recorded. A further limitation in the present study is that while participants’ age, gender and level of learning difficulty was representative of the entire school population, the sample contained a lower proportion of children with profound and multiple learning disability. The results, therefore, may not fully translate to children with more profound learning disabilities.

Children with developmental disability are likely to have ongoing health-related issues which require them to attend multiple appointments. Providing eyecare in-school removes the organisational burden of arranging this appointment, something which was reported as an additional benefit by parents; *“it is helpful for parents to have this in school as many parents have a lot of appointments to attend*,*” “providing this test within school saves my time and hassle making appointments outside*,*” “one less appointment for parents to chase after*.*”* The convenience for parents of providing eyecare in-school was one of the top-rated benefits reported by both parents and teachers, with two parents commenting *“very beneficial as I am a working mum*” and *“very convenient for working parents*.

Compliance with assessments was high during the in-school eye examination, with over 90% success rate achieved for the majority of tests [13]. In contrast, parents reported limited compliance with assessments at previously accessed eyecare services; *“my son tends to get very stressed as he is asked to do a lot in a short space of time*. *Tests tend to be inconclusive due to lack of cooperation*.*”* The added benefit of providing eyecare in-school allows the examination to be carried out over multiple, shorter visits if necessary in order to complete a conclusive eye examination. In the present study almost one third of children required at least two visits to complete the testing procedures [13]. These ‘mop up’ visits fitted easily into the in-school testing schedule and is echoed in previous work [5]. The in-school setting also allowed the clinicians to collaborate with teaching staff to identify the most appropriate time to examine the child based on their behavioural and emotional needs.

Disruption of class activities due to attending the eye examination was not considered problematic, with majority of teachers reporting that the eyecare appointments were not disruptive to routine class activities. In fact, several parents commented that provision of eyecare at school resulted in the child having less time out of school to attend external appointments; *“it was beneficial to receive this eye examination at school rather than being removed from school and going to the hospital environment*,*”* and *“it is a good idea to have this done in school–no hassle*, *no change of environment and less time away from school for appointments*.*”*

### Vision report

Both parents and teachers reported positively on the value and usefulness of the written Vision Reports. Given that jargon-free reporting of visual status is valued and used by recipients and verbal information has been shown to be poorly retained at clinic appointments [10, 11], meaningful reporting of visual status should be integral to in-school eyecare services. Without this component, services will fail to impact optimally on children’s visual and educational outcomes. This has been evidenced through previous work from our group; 27.5% of children (n = 55) had at least one unmet visual need at baseline which required environmental modifications and advice only, which was detailed in the Vision Report (i.e. unmet needs were not due to lack of refractive correction). At follow-up, this number reduced to 9.0% (n = 18) [13]. Without provision of the written report, parents and teachers would have been unable to implement the required modifications to address these children’s visual needs.

Teachers valued and acted on information regarding their pupil’s vision and visual needs. One teacher commented that the Vision Report *“can impart information relating to environmental factors that can influence work/activities relating to pupils*.*”* Donaldson et al. [5] report that the regular presence of eyecare professionals in special school settings allows for more effective dissemination of relevant information to teaching staff and has the added benefit of raising awareness of vision among staff [5]. In the present study, 83% of teachers valued the opportunity to speak directly to the eyecare provider regarding a child’s vision, further highlighting the importance of increased communication between educators and clinicians. Parents found the Vision Report useful, but many failed to fully implement recommendations included in the report, indicating that parents may require assistance in adapting a child’s environment to compensate for visual deficits. Directing parents and teachers to vision support and habilitation services may be a useful supplement to written information, for example referral to a Qualified Teacher of the Visually Impaired if a child meets the criteria for this service.

Parents were generally unsure whether teachers had implemented modifications to the school environment as suggested in the Vision Reports despite 80% of teachers reporting they had implemented the suggested actions in the report. This finding suggests that communication between parents and teachers was not optimal. Lehman [14] highlights the importance of good communication between stakeholders involved in the child’s care and notes that specific advice, including adjustments to the child’s environment and visual materials, should be well documented and made available to educators to allow incorporation into the child’s Individual Educational Plan (IEP) [14]. This encourages a joined-up approach to the child’s care and ensures visual difficulties are accounted for to maximise children’s access to the curriculum. In the present study, one teacher commented *“it is a worthwhile idea to make teachers/parents aware of visual problems of the children and how to provide a better environment for them*,*”* while another stated *“information gathered can be shared with the teacher for the benefit of the child*.*”*

### Vision information on statutory educational documents

A SEN/EHCP is a statutory document which includes a description of a child’s needs and outlines additional help required to meet these needs in an educational setting [24]. At the beginning of the statementing process, healthcare professionals involved in the care of the child (e.g. speech and language therapy, paediatrician) are often contacted to provide clinical information which will feed into the SEN/EHCP. Little and Saunders [25] carried out a review of SEN/EHCPs and compared the vision-related detail they contained with clinical notes [25]. This comparison revealed that vision information on statutory documents was often inadequate or entirely lacking for children with significant visual needs. Outputs from the present study concur with this report. The SEN/EHCPs examined in the present study reveal that, where present, visual information was limited to stating the presence of a deficit, with no articulation of the practical or educational implications (if any) of these visual characteristics, nor how a teacher might adapt the child’s educational environment to account for the visual deficit and improve access to the curriculum. For example, one child’s statement identified that the child has *“moderate alternating convergent squint”* and *“visual acuity of 6/48*.*”* Generic advice was provided in this child’s statement, but nothing which articulated how this deficit would impact in the learning environment or inform educators regarding the size of learning material that would be visible to the child. Input from a Qualified Teacher of the Visually Impaired may be warranted to provide practical advice in this instance, however not all children with a vision deficit will meet the criteria for this service. A child’s SEN/EHCP is reviewed annually by the school, parents and Education Authority and is updated where significant amendments are required. The document remains with the child throughout formal education. It is clearly vital that accurate, up-to-date, relevant and specific information is included on statutory documents, allowing educators to make appropriate adjustments in the classroom to ensure visual limitations do not impinge on children’s ability to access the curriculum.

## Conclusions

Parents and teachers reported positively on the provision of comprehensive in-school eyecare for children in special schools, with parents showing preference for this service compared to previously accessed eyecare services. Jargon-free, written reports of visual status are valued and utilised by parents and teachers, and help ensure children are receiving appropriate vision-related modifications to home and school environments. Parents and teachers may benefit from further support in making vision-related adjustments for the children in their care. Currently, vision information on statutory documentation, where present, lacks specific advice and guidance to allow educators to ensure children are able to access the curriculum.

## Supporting information

S1 Dataset (XLSX)

S1 Fig Example Vision Report. (PDF)

S2 Fig Feedback questionnaire issued to parents. (PDF)

S3 Fig Feedback questionnaire issued to teachers. (PDF)
