# Supplementary material for: Parents and teachers of children in special education settings value in-school eyecare and written reports of visual status
Source: PLoS One. 2020 Sep 11;15(9):e0238779. doi: 10.1371/journal.pone.0238779 (PMC7485870; doi:10.1371/journal.pone.0238779)
Supplement: S1 Fig — (PDF) [file pone.0238779.s002.pdf]

Section 1 - Details of child	
Child's name	Declan
D.O.B	Age 4 years
School	FP
Date of test	30th May 2017

Results of your child's vision assessment

We hope the following information is useful. We have used the information you gave us about your child and the results we obtained when testing their eyes, to describe their vision.

Section 2 - Additional detail about the eye test	
Who was present at the eye test?	Classroom assistant present, parent unable to attend.
What was already known about eyes and vision?<br>Did anyone have questions about eyes and vision?	Declan has Down syndrome and has had glasses from local hospital eye clinic for over a year. Next appt for hospital eye clinic 4 months. Poor compliance with specs. Nystagmus (wobbly eyes) noted soon after birth. Investigated at hospital – no action required. Nystagmus less obvious now than in infancy. Teacher/parents not sure how good vision is. No major concerns re vision.

Section 3 – Summary: The child's eyes and vision

Declan worked hard today and was able to cooperate with most of the tests we tried. He has had glasses for some time now but doesn't wear them very well. Declan needs to wear his glasses all the time to make sure his vision is as good as it can be. Please help Declan wear his glasses by making him feel special and that everyone is pleased with him when he keeps them on. We have discussed methods to try and encourage glasses wear with his teacher, who will try and work with him at school. We have enclosed a leaflet which provides some strategies to try. This is also available as a video animation at the following link: https://www.youtube.com/watch?v=hUcvYm19NGQ. It may take a while before full-time wear is achieved, but keep trying!

Even with his glasses on Declan's vision at low and high contrast is reduced compared to other children his age. Reduced vision is common in Down syndrome and with nystagmus (wobbly eyes) but needs to be considered in the classroom or he will miss out on important information. Declan sees best when he tips his chin down and looks up at things because his eyes wobble less in this position. He needs round spectacle frames and a tilted desk at school to make the most of this useful habit - please don't discourage it. Declan is also likely to concentrate better and see better if not too much information is presented on a page. See further in the report for more information on how to achieve this.

Actions from today's test:

Glasses needed	YES
Modifications to classroom/ schoolwork needed	YES
Statement of Educational Need should include information about vision needs	YES

Reading and writing materials need to be larger and bolder I have included examples of the size of letter/pictures/PECs Declan can easily see.
A soft dark pencil (e.g. 8B) or a black marker pen should help Declan with writing/drawing.
Black on white information will be more visible than grey or pale colours on white.
Presenting one thing at a time (isolated presentation) is important
Declan needs to sit close to the smartboard and be allowed to get close to what he needs to see. A tilted desk may help him make best use of his vision for near work.

Child is eligible for certification as visually impaired NO

GP Action required NO

Another specialist needs to see this child YES

It would be helpful for a Qualified Teacher for the Visually Impaired (QTVI) or Vision Support teacher to see Declan and help his teachers make modifications to his school work and environment.

Section 4 – We tested to see if glasses are needed

This was tested: Yes ☒ No ☐ This was difficult to assess today ☐

We measured for **focusing accuracy**:

This was tested: Yes ☒ No ☐ This was difficult to assess today ☐

Details: Focussing is accurate with glasses on.

We gave a new prescription for glasses: Yes ☒ No ☐

Details: Declan is long-sighted with astigmatism. He should wear his glasses all the time to ensure he has the best possible vision when looking both close up and far away. Advise round eye shape as vision best on upgaze.

Declan's new prescription:
R +4.00/-2.00x180 L +4.50/-2.50x180

Section 5 – Results of the vision tests we did today

Visual acuity: describes how well a person sees black on white detail with glasses if needed.

We were able to measure visual acuity for looking at things:

in the distance ☒ close up ☒ both were difficult to test today ☐

Vision (with old glasses) 6/19 – both eyes open using Cardiff preferential looking cards.

We used a picture test to see how well Declan can see. He would only cooperate with both eyes open as he was uncomfortable having either eye covered. Declan's vision is lower than we would expect for a child his age. He will benefit from using enlarged printed and electronic material, and moving closer to objects he is looking at. We have included examples of what he should be able to see clearly.

Binocular vision and eye movements: This is how well your child's eyes work together

This was tested today: Yes ☒ No ☐ This was difficult to assess today ☐

Details: Declan has nystagmus – his eyes wobble slightly when he is looking at things. The wobble has been investigated in the past by the hospital eye service and has got a little less over time. However, Declan's vision is not as good as expected for his age and the nystagmus is part of the problem. Declan's eyes wobble a bit less when he tips his chin down and looks up at things.

Visual Field: This is how well your child can see things to the side of their central vision

This was tested today: Yes ☒ No ☐ This was difficult to assess today ☐

Details: Declan's peripheral (side) vision appears normal.

Contrast Sensitivity: This is how well objects are seen against different backgrounds

This was tested today: Yes ☒ No ☐ This was difficult to assess today ☐

Details: Declan's low contrast vision was below expected for his age. This means that he may have some difficulty seeing pictures/words if they are a similar colour or shade to the background e.g. pale blue writing on a white background. It is important for Declan that the information on the whiteboard, and in his other school work, is high contrast (e.g. black on white, black on yellow) and that he has good lighting when doing his school work. It may be useful for Declan to use a thick, dark pen/marker when writing or drawing as this should be easier for him see.

Evidence of Visual Processing difficulties: This is when there are visual difficulties caused by problems interpreting visual information in the brain rather than the eyes.

This was tested today: Yes ☒ No ☐ This was difficult to assess today ☐

Using the visual skills inventory, Declan's mum has highlighted a number of difficulties with how Declan processes information in a busy or crowded environment.

Children who have such difficulties may find a 'crowded' visual scene difficult to interpret. For example, when trying to find an item in a toy box, Declan may struggle if the item he is looking for is surrounded by other items, or is on a patterned surface.

Children with such difficulties often perform better when work (food, school work, craft etc.) is placed on a plain surface and when distracting 'extra' items are removed from the work surface. Plain duvet covers, carpets, tablecloths, walls and plates may all help reduce visual distraction and make these objects easier for Declan to see and process. Keeping his environment tidy and clear from clutter should also help with this. Using storage boxes to store his personal items and toys at home is an easy way to do this. Minimising photos on the wall, patterned wallpaper and carpets etc. will minimise clutter and distracting visual information.

It may be easier for Declan to sit close to whatever he is looking at, e.g. TV, family members, school board etc. When taking Declan to the shopping centre or supermarket, it may be easier to take her during quieter, off-peak times to help him feel more at ease and relaxed.

Section 6 – Results of the eye health check

This was tested today: Yes ☒ No ☐ This was difficult to assess today ☐

Does the child need to see another specialist about their eye health? Yes ☐ No ☒

Details: Both Declan's eyes appear healthy.

Section 7 – Technical details for other health professionals		
**Visual Acuity**	*Cardiff acuity test at 50cm*	With current Rx:<br>Binoc 6/19 – reduced for age. No co-operation with monoc VA.
**Refractive Error**	*With cycloplegia*	R +4.00/-2.00x180 L +4.50/-2.50x180<br><br>Given full plus spectacles for full-time wear. Monitor need for bifocal in future but note effect of upgaze on nystagmus – contraindication for bifocal if persistent.
**Accommodative Function**	*Dynamic retinoscopy*	Accurate with glasses, significant lag unaided
**Ocular Posture and Eye Movement**	*Cover test, ocular motility*	Fine manifest-latent nystagmus in primary position – reduced on up gaze. No manifest strabismus. Ocular motility grossly full.
**Contrast**	*Cardiff contrast test*	12.5 (8%contrast) – reduced for age
**Visual Field**	*Gross confrontation*	Grossly full and symmetrical
**Eye Health Exam**	*Binocular indirect through dilated pupils*	External eye clear R&L; Media clear R&L; fundi healthy R&L; healthy discs.
**Stereopsis**	*Frisby*	Equivocal to grossest target– revisit next time.
**Visual Processing**	*Visual skills inventory*	See section 5

Section 8: Assessors

Who is this report from?

Name: Mr A Optom

Role: Optometrist

Address:

Who is getting a copy of this report? Parents, Teacher.
