# Supplementary material for: Parents and teachers of children in special education settings value in-school eyecare and written reports of visual status
Source: PLoS One. 2020 Sep 11;15(9):e0238779. doi: 10.1371/journal.pone.0238779 (PMC7485870; doi:10.1371/journal.pone.0238779)
Supplement: S2 Fig — (PDF) [file pone.0238779.s003.pdf]

Evaluating In-School Vision Testing: How was it for you?

Parent Questionnaire

We would like to thank you again for allowing your child to take part in **The SEE Project**. As you know, we have been offering in-school eye examinations to pupils in special educational schools as part of a research study. We hope that this research will be helpful in planning future eye care services for children in special education.

This is your chance to tell us what you think eye care services for children in special education should be like and to tell us what you think of the in-school eye examination your child had by filling in this short survey***. We would be very grateful for your feedback, comments and suggestions.*** This questionnaire should take 5-10 minutes to complete.

By completing this questionnaire we are assuming that you are giving your consent to participate in this study, however you are free to withdraw at any time. Once completed please return the questionnaire to the school in the envelope provided.

Ethical approval has been granted by the Research Ethics Committee at Ulster University.

Child's initials ______ Child ID_____

1. **How useful do you think the in-school eye test was for you, your child and school staff?**

	Not at all useful	Somewhat useful	No strong opinion	Useful	Very useful
You	1	2	3	4	5
Your child	1	2	3	4	5
School staff	1	2	3	4	5

2. **Please rate your experience of the following items regarding in-school eye tests:**
   1= very poor, 2= poor, 3= no strong opinion, 4= good, 5 = very good, 6=don't know

   Convenience
   1 2 3 4 5 6

   Flexibility of appointment times
   1 2 3 4 5 6

   Written communication of test results
   1 2 3 4 5 6 ☐ didn't receive any

   Other comments ____________________

3. **In your opinion, do any of the following benefits or limitations apply to in-school eye tests?** *Please tick any that you think apply.*

Benefits

- ☐ Familiar environment for my child
- ☐ Convenient for parents
- ☐ Parent may not be present
- ☐ Testing can be carried out over multiple short visits if required
- ☐ Other classmates taking part encourages compliance
- ☐ Teacher can ask eye care provider questions directly about child's vision
- ☐ Increases awareness of vision among teachers
- ☐ Child cooperates better for school staff

Other ____________________

Limitations

- ☐ Child misses class activities during eye test
- ☐ Parent may not be present
- ☐ Blurred vision from drops disrupts school work
- ☐ Disrupts school routine
- ☐ Unsettling for child
- ☐ Unable to ask eye care provider questions about my child's eyes at the time of test

Other ______________________________

4. **If your child had an eye test in school and needed to get glasses, where would you like to get the glasses from?**
   - ☐ I would be happy for my child to get the glasses at school – I don't mind if I don't choose the glasses
   - ☐ I would be happy for my child to get the glasses at school – as long as I could help choose the glasses
   - ☐ I would prefer to take my child to the local opticians to get glasses
   - ☐ I have no preference

5. **If your child has previously had their eyes tested at <u>the hospital</u> please rate your experience of the following items:** 1= very poor, 2= poor, 3= no strong opinion, 4= good, 5 = very good, 6= don't know

Convenience of attending appointment

1 2 3 4 5 6

Flexibility of appointment times

1 2 3 4 5 6

Written communication of test results

1 2 3 4 5 6 ☐ I didn't receive any

Other ______________________________

6. **If your child has previously had their eyes tested at <u>the local opticians</u> please rate your experience of the following items:** 1= very poor, 2= poor, 3= no strong opinion, 4= good, 5 = very good, 6=don't know

Convenience of attending appointment
1 2 3 4 5 6

Flexibility of appointment times
1 2 3 4 5 6

Written communication of test results
1 2 3 4 5 6 ☐ I didn't receive any

Other ______________________________

The following questions relate to the report we sent you after we tested your child's eyes in school.

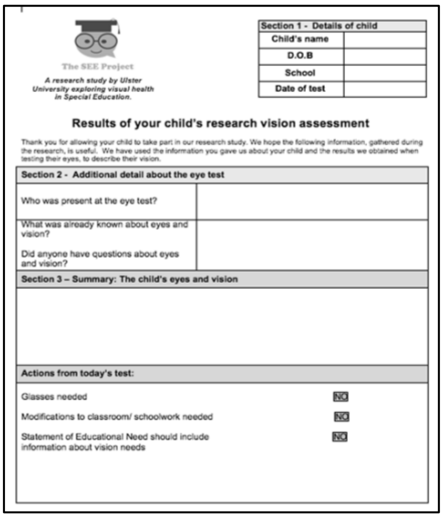

The SEE Project

A research study by Ulster University exploring visual health in Special Education.

Section 1 - Details of child	
Child's name	
D.O.B	
School	
Date of test	

Results of your child's research vision assessment

Thank you for allowing your child to take part in our research study. We hope the following information, gathered during the research, is useful. We have used the information you gave us about your child and the results we obtained when testing their eyes, to describe their vision.

Section 2 - Additional detail about the eye test	
Who was present at the eye test?	
What was already known about eyes and vision?<br>Did anyone have questions about eyes and vision?	
**Section 3 – Summary: The child's eyes and vision**	
**Actions from today's test:**	
Glasses needed	NO
Modifications to classroom/ schoolwork needed	NO
Statement of Educational Need should include information about vision needs	NO

7. **Did you receive a report for your child?**

☐ Yes ☐ No *If no, please go to question 16.*

8. **Did you read the report?** *(Don't worry if you haven't read the report – this is useful for us to know).*

☐ Yes ☐ No

If no, please tell us why you didn't read it, then go to question 16

____

____

9. **Is the information contained in the report useful on a day-to-day basis?**

Not at all useful	Parts are useful	No strong opinion	Quite useful	Very useful
1	2	3	4	5

10. **What were the *<u>most</u>* helpful parts of the report?**

____

____

11. **What were the *<u>least</u>* helpful parts of the report?**

____

____

12. **Was the information in the report written in a way you could understand?**

Difficult to understand	Somewhat difficult	No strong opinion	Fairly easy	Easy to understand
1	2	3	4	5

13. **Did the report contain any information about your child's eyes and vision that you didn't know about before?**

☐ Yes ☐No ☐ Not sure

If yes, please provide details.

14. **If the report contained any action points or modifications (e.g. wearing glasses full time, needing large print material etc.) relating to how your child could best use their vision, have any adaptations been made or planned:**

a) at home?

☐ Yes ☐No ☐ Don't know ☐ Not applicable

Please comment on any modifications that have been made, or reasons why modifications have not been made.

b) at school?

☐ Yes ☐No ☐ Don't know ☐ Not applicable

15. **a) Does your child's Statement of Educational Need (SEN) include information about your child's eyes or vision?**

☐ Yes ☐ No ☐ Don't know ☐ Not applicable

b) Do you think it is important to include information about your child's eyes or vision in their SEN?

☐ Yes ☐ No ☐ No strong opinion

16. **Please use the space below to make any other comments about The SEE Project. We would welcome your feedback on the project and value any suggestions about how it can be improved in the future.**

Thank you for taking the time to complete this questionnaire.
