# Supplementary material for: Parents and teachers of children in special education settings value in-school eyecare and written reports of visual status
Source: PLoS One. 2020 Sep 11;15(9):e0238779. doi: 10.1371/journal.pone.0238779 (PMC7485870; doi:10.1371/journal.pone.0238779)
Supplement: S3 Fig — (PDF) [file pone.0238779.s004.pdf]

Evaluating In-School Vision Testing: How was it for you?

Teacher Questionnaire

We would like to thank you again for taking part in **The SEE Project**. As you know, we have been offering in-school eye examinations to pupils in special educational schools as part of a research study. We hope that this research will be helpful in planning future eye care services for children in special education.

As part of the research we would like to find out your opinion of the in-school vision testing by completing this short questionnaire. We would be very grateful for your feedback, comments and suggestions. This questionnaire should take 5-10 minutes to complete.

By completing this questionnaire we are assuming that you are giving your consent to participate in this study, however you are free to withdraw at any time. Once completed please return to The SEE Project postbox in the envelope provided.

Ethical approval has been granted by the Research Ethics Committee at Ulster University.

Class ________

1. **How useful do you think the in-school eye tests were for the pupils, school staff and parents?**

	Not at all useful	Somewhat useful	No strong opinion	Useful	Very useful
Pupils	1	2	3	4	5
Staff	1	2	3	4	5
Parents	1	2	3	4	5

2. **To what extent do you feel the eye tests disrupted the pupils' other school activities?**

Disrupted a lot		No strong opinion		Did not disrupt at all
1	2	3	4	5

3. **In your opinion, do any of the following benefits or limitations apply to in-school eye tests?** *Please tick any that you think apply.*

Benefits

- ☐ Familiar environment for child
- ☐ Convenient for parents
- ☐ Parent may not be present
- ☐ Testing can be carried out over multiple short visits if required
- ☐ Other classmates taking part encourages compliance
- ☐ Teacher can ask eye care provider questions directly about child's vision
- ☐ Increases awareness of vision among teachers

Limitations

- ☐ Pupils miss class activities during eye test
- ☐ Parent may not be present
- ☐ Not enough staff to accompany pupils
- ☐ Blurred vision from drops disrupts school work
- ☐ Disrupts school routine
- ☐ Unsettling for child
- ☐ Lack of space available to carry out eye tests

Other ____

The following questions relate to the report provided describing your pupils' visual status following their sight test.

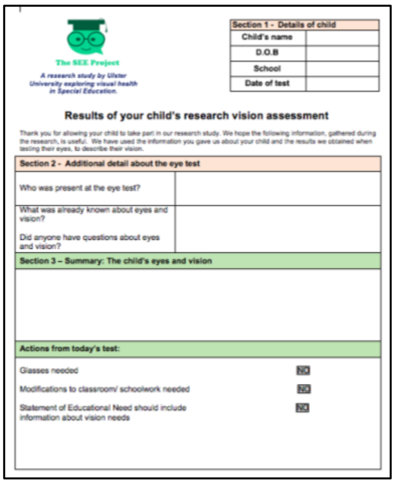
The SEE Project

A research study by Ulster University exploring visual health in Special Education.

Section 1 - Details of child	
Child's name	
D.O.B	
School	
Date of test	

Results of your child's research vision assessment

Thank you for allowing your child to take part in our research study. We hope the following information, gathered during the research, is useful. We have used the information you gave us about your child and the results we obtained when testing their eyes, to describe their vision.

Section 2 - Additional detail about the eye test	
Who was present at the eye test?	
What was already known about eyes and vision?<br>Did anyone have questions about eyes and vision?	

Section 3 – Summary: The child's eyes and vision

Actions from today's test:

Glasses needed	NO
Modifications to classroom/ schoolwork needed	NO
Statement of Educational Need should include information about vision needs	NO

4. Did you receive a report for any of your pupils?

☐ Yes ☐ No *If no, go to question 13.*

5. Did you read the report(s)? *(Don't worry if you haven't read the report – this is useful for us to know).*

☐ Yes, straight away. ☐ No, but I plan to during the holidays.

☐ Yes, several weeks later. ☐ No, I have not read the reports.

☐ Yes, several months later. Month read (if known) ____________

If no, please tell us why you didn't read them, then go to question 13

____

____

6. Did you find the information in the report(s) useful and relevant to your work with the pupil(s)?

Not at all useful	No strong opinion	Parts are useful	Quite useful	Very useful
1	2	3	4	5

7. Was the information contained in the report(s) written in a way you could understand?

Difficult to understand	Somewhat difficult	No strong opinion	Fairly easy	Easy to understand
1	2	3	4	5

8. What were the *most* helpful parts of the report(s)?

____

____

10. What were the *least* helpful parts of the report(s)?

____

____

11. a) If the report(s) contained any recommendations relating to how the pupil(s) could best use their vision in the classroom, have any adaptations (e.g. classroom position, enlarging text size) been made or planned? *Please provide details.*

☐ Yes ☐ No ☐ Not sure

____

____

b) If a child in your class has a vision problem, do you feel confident implementing suggested classroom modifications?

☐ Yes ☐ No ☐ Not sure

c) Would you be interested in having further training to help you learn how to adapt a child's learning environment/materials if they have a vision problem?

☐ Yes ☐ No ☐ Not sure

12. **Do you think the pupils' Statement of Educational Need should include details from the report(s) if they highlight a visual problem?**

☐ Yes ☐ No ☐ No strong opinion

13. **Please use the space below to make any other comments about The SEE Project. We would welcome your feedback on the project and value any suggestions about how it can be improved in the future.**

____

____

____

Thank you for taking the time to complete this questionnaire.
